# Double pass 595‐nm dye laser is not more effective than single pass: A systematic review and meta‐analysis

**DOI:** 10.1111/srt.13237

**Published:** 2022-12-19

**Authors:** Lingling Yang, Xiaoyu Sun, Wenzhong Xiang

**Affiliations:** ^1^ Department of Dermatology, Affiliated Hangzhou Dermatology Hospital Zhejiang University School of Medicine Hangzhou China

Dear Editor,

Port wine stain (PWS) is a congenital capillary malformation, affecting 0.3%–0.5% of the population.^1^ It is characterized by a single pink and flat patch in infancy and may become thicken and darken during adults life, which significantly disturb the patients’ quality of life. Puled dye laser (PDL) has become the gold standard treatment of PWS.^2^, ^3^ However, there are only a minority of patients achieving total clearance of the PWS.^4^ To improve the clearance rate of PWS, the concept of double‐pass pulsed‐dye laser (DPL) and single‐pass pulsed‐dye laser (SPL) were introduced. Nevertheless, compared to SPL, DPL has shown variable results.^5^, ^6^, ^7^, ^8^, ^9^, ^10^ The current position of DPL and SPL in the therapeutic scheme of PWS is lacking evidence‐based recommendations on efficacy and side effect. This meta‐analysis aims to compare the efficacy and tolerability of DPL versus SPL.

The study protocol was preregistered on PROSPERO (CRD42022299613). PUBMED, EMBASE, Web of Science and Cochrane library of randomized Controlled Trials were searched without any date restrictions. In addition, we searched the reference articles of eligibility studies reported. We excluded articles involving the use of dye laser combined with other laser equipment or topical drugs. Lingling Yang and Xiaoyu Sun independently extracted data from included studies. Four outcomes were used to compare the effect and safety: better clearance rate, visual improvement scale, blanching rate, and side effect rate. The meta‐analysis was performed with the Review Manager software (RevMan 5.3.5). We assessed the risk of bias of randomized controlled trials (RCTs) by The Risk of Bias Tool. Funnel plots were assessed to detect potential publication bias. We calculated *I* ^2^ statistics to examine the level of Heterogeneity. And, we did an additional sensitivity analysis with respect to trials included.

Six trials comprising 130 patients were included after full‐text screening. Study characters can be found in Table 1. The pooled analysis revealed no significant difference between DPL and SPL in blanching rate (MD = 0.04, 95% CI: −0.07 to 0.16, P = 0.46), better clearance rate (RR = 1.60, 95%CI:0.80 to 3.22, P = 0.19) and visual improvement (MD = 0.04, 95%CI:−0.28 to 0.36, *p* = 0.81). Furthermore, there was no difference in the side effect between two groups (RR = 1.17, 95% CI: 0.60 to 2.28, *p* = 0.65) (Figure 1). We used fixed‐effects model when the statistical heterogeneity was not important. The random‐effects model was used if the statistical heterogeneity was moderate. All of the pooled effects and side effects remain relatively stable in sensitivity analyses.

**TABLE 1:** Characteristics of included studies

Author Year of publication (location)	Participants characteristics	Study design	Intervention group (DPL)	Control group (SPL)	Treatment schedule	Dropout rate	Side effect
Noormohammad 2021 (Iran)	24	Double‐blind Randomized evaluator‐blinded control trial	7 mm, 20 ms, 13 J/cm^2^ +7 mm, 1.5 ms, 11 J/cm^2^ (at a 20‐min interval)	7 mm, 1.5 ms, 11 J/cm^2^	3 sessions 1 months interval	0%	DPL: 2 hyperpigmentation SPL: 1 hyperpigmentation
Yu 2018 (China)	21	Randomized, single‐blinded, self‐control‐led comparative study	10 mm, 1.5 ms,10 J/cm^2^; 7 mm, 1.5 ms, 12 J/cm^2^+ 10 mm, 1.5 ms, 9 J/cm^2^; 7 mm, 1.5 ms, 11 J/cm^2^ (at a 30‐min interval)	10 mm, 1.5 ms, 10 J/cm^2^; 7 mm, 1.5 ms, 12 J/cm^2^	3 sessions 2 months interval	0%	DPL: 3 hyperpigmentation SPL: 3 hyperpigmentation
Sajjad 2020 (Pakistan)	30	Single blind‐randomized controlled tria	7 mm, 2–6 ms, 8‐12 J/cm^2^ +7 mm, 2‐6 ms, 4‐6 J/cm^2^ (at a 30‐min interval)	7 mm,2‐6 ms, 8–12 J/cm^2^	4 sessions 3 weeks interval	0%	No side effect
Peter 2012 (Netherlands)	17	Randomized evaluator‐blinded trial	7 mm, 1.5 ms 11 J/cm2 × 2 (at a 6‐min interval)	7 mm,1.5 ms 12 J/cm^2^	2 sessions 6–10 weeks interval	5.9%(1) (personal reasons)	DPL: 4 hyperpigmentation 2 hypopigmentation 1 scar SPL: 4 hyperpigmentation 2 hypopigmentation
David 2008 (Britain)	18	Randomized Controlled trial	7 mm, 0.45 ms, 7 J/cm2 × 2 (at a 30‐s interval)	7 mm, 0.45 ms, 7 J/cm^2^	1 session	11.1% (2) (personal reasons)	No side effect
Brauer 2017 America	20	single‐center, randomized, Controlled, split‐lesion pilot study	10 mm, 0.45–1.5 ms 6–9 J/cm^2^+ 10 mm, 6 ms 7.5 J/cm^2^ (at a 2‐day interval)	10 mm, 0.45–1.5 ms 6–9 J/cm^2^	2 sessions 4–8 weeks interval	0%	DPL: 1 Hypopigmentation 1 hyperpigmentation SPL: 1 Hypopigmentation 1 hyperpigmentation

**FIGURE 1:**
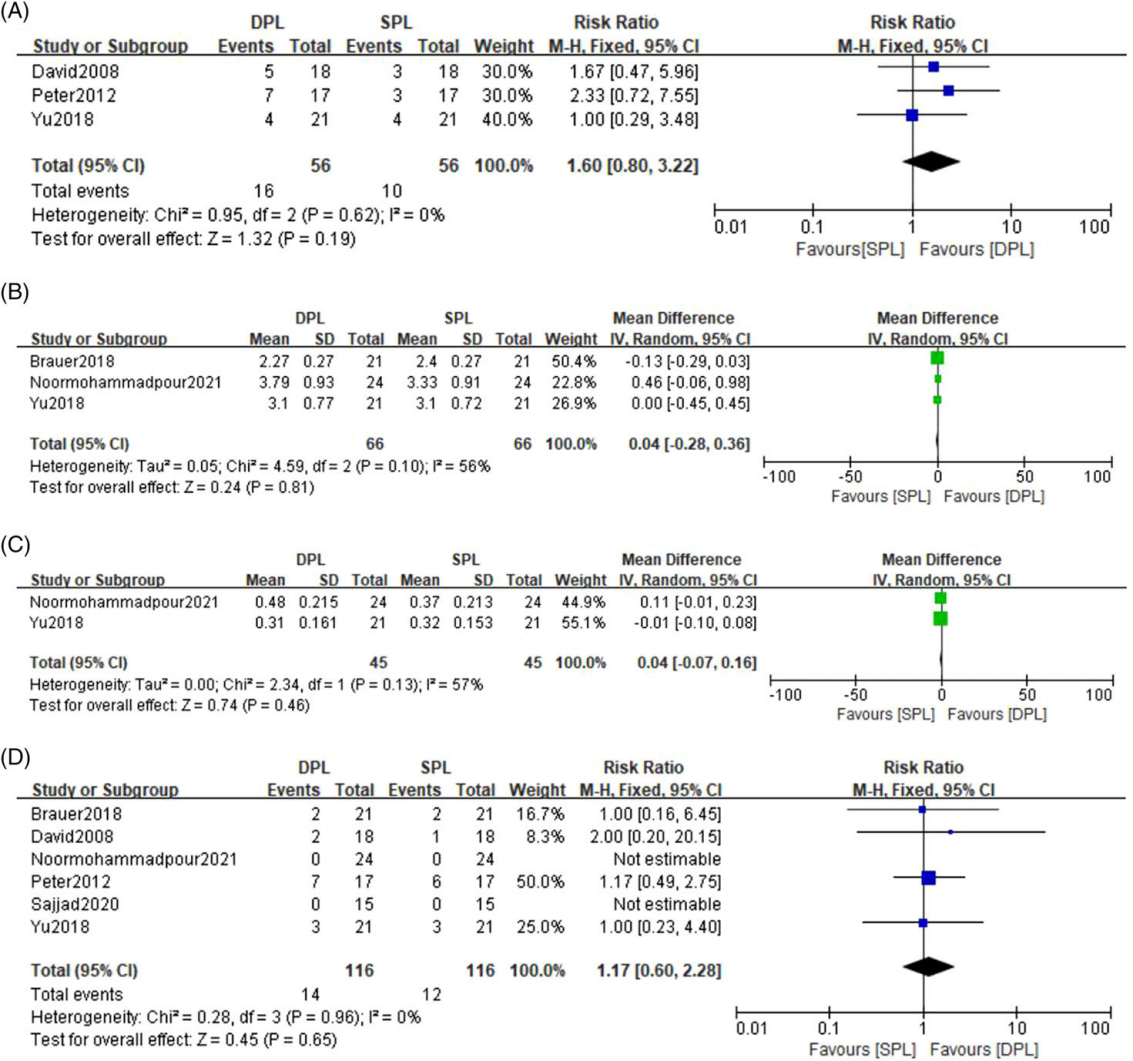
Forest plot revealed that there were no significant difference between double‐pass pulsed‐dye laser (DPL) and single‐pass pulsed‐dye laser (SPL) in better clearance rate (A), visual improvement (B), blanching rate (C), and side effect (D).

The current evidence demonstrated that DPL had similar efficacy and side effects with SPL. There are multiple possible reasons for the similar results. First, skin lesion depth of PWS ranges from 1 to 5 mm.^11^ But, PDL had limited ability to affect vessels residing deeper than 2 mm in the skin^12^, ^13^ Double pass depth of vascular injury was 2.78 ± 0.9 mm for inter‐pulse interval of 30 min, which was significantly greater than SPL (0.9 ± 0.2 mm),^14^ but this therapy was conducted on normal skin, not PWS lesions. Another trail's results indicated photothermal damage of a similar depth after SPL and DPL therapies, with mildly greater vessel wall damage on the DPL‐treated sides.^7^ Therefore, whether the depth of vascular injury in DPL increases is unknown. Second, Previous studies had shown that angiogenesis pathways (specifically vascular endothelial growth factor and hypoxia inducible factor‐1α) were activated during the wound healing process.^15^, ^16^, ^17^ Compared to SPL, the more serious injury of DPL may lead to more postlaser revascularizations, indicating the limited effect of PDL.

This study had a few limitations. First, three randomized studies had a high risk of bias; two randomized trails had a moderate risk of bias. However, our analysis included only prospective, controlled studies to capture the evidence available. Second, the total number of studies and patients with PWS was low. Nonetheless, all the included trails were RCTs.

In conclusion, at present, PWS cannot be cured completely, although PDL was considered as the gold standard treatment. This combined systematic review demonstrated that the effectiveness of DPL was comparable to SPL.

## CONFLICT OF INTEREST

The authors declare that there is no conflict of interest that could be perceived as prejudicing the impartiality of the research reported.

## Data Availability

Research data are not shared.
